# Cellular Stress Induced Alterations in MicroRNA let-7a and let-7b Expression Are Dependent on p53

**DOI:** 10.1371/journal.pone.0024429

**Published:** 2011-10-11

**Authors:** Anthony D. Saleh, Jason E. Savage, Liu Cao, Benjamin P. Soule, David Ly, William DeGraff, Curtis C. Harris, James B. Mitchell, Nicole L. Simone

**Affiliations:** 1 Radiation Oncology Branch, National Cancer Institute, National Institutes of Health, Bethesda, Maryland, United States of America; 2 Translational Medicine Branch, National Heart Lung and Blood Institute, National Institutes of Health, Bethesda, Maryland, United States of America; 3 Laboratory of Host Defenses, National Institute of Allergy and Infectious Diseases, National Institutes of Health, Bethesda, Maryland, United States of America; 4 Radiation Biology Branch, National Cancer Institute, National Institutes of Health, Bethesda, Maryland, United States of America; 5 Laboratory of Human Carcinogenesis, National Cancer Institute, National Institutes of Health, Bethesda, Maryland, United States of America; University of Kentucky College of Medicine, United States of America

## Abstract

Genotoxic stressors, such as radiation, induce cellular damage that activates pre-programmed repair pathways, some of which involve microRNAs (miRNA) that alter gene expression. The let-7 family of miRNA regulates multiple cellular processes including cell division and DNA repair pathways. However, the role and mechanism underlying regulation of let-7 genes in response to stress have yet to be elucidated. In this study we demonstrate that let-7a and let-7b expression decreases significantly following exposure to agents that induce stress including ionizing radiation. This decrease in expression is dependent on p53 and ATM *in vitro* and is not observed in a p53^−/−^ colon cancer cell line (HCT116) or ATM^−/−^ human fibroblasts. Chromatin Immunoprecipitation (ChIP) analysis showed p53 binding to a region upstream of the let-7 gene following radiation exposure. Luciferase transient transfections demonstrated that this p53 binding site is necessary for radiation-induced decreases in let-7 expression. A radiation-induced decrease in let-7a and let-7b expression is also observed in radiation-sensitive tissues *in vivo* and correlates with altered expression of proteins in p53-regulated pro-apoptotic signaling pathways. In contrast, this decreased expression is not observed in p53 knock-out mice suggesting that p53 directly repress let-7 expression. Exogenous expression of let-7a and let-7b increased radiation-induced cytotoxicity in HCT116 p53^+/+^ cells but not HCT116 p53^−/−^ cells. These results are the first demonstration of a mechanistic connection between the radiation-induced stress response and the regulation of miRNA and radiation-induced cytotoxicity and suggest that this process may be a molecular target for anticancer agents.

## Introduction

Radiation-induced cellular damage results in a stress response that involves large scale changes in gene expression including that of miRNA [1], [2], [3]. miRNA comprise a highly conserved family of small (18–24 nucleotide) non-coding RNAs that bind to mRNA molecules and post transcriptionally regulate expression [4]. Reduced expression of the let-7 miRNA family has been shown to be activated in response to irradiation [2], [3]. Deletion or mutation of let-7 family expression is highly associated with the development of cancer [5], while the presence of increased let-7b decreases lung tumor growth in mice [6] and sensitizes lung cancer cells to radiation [2]. This is in part explained by the fact that let-7 family members have been shown to target expression of proteins involved in cell proliferation such as Ras [7] and cell cycle regulation such as Cdc25A and cyclin D1 [8], [9], which contribute to its tumor suppressor phenotype. However, the mechanism underlying the decrease in let-7 expression after irradiation has not yet been elucidated, and a greater understanding may allow for manipulation of these pathways for therapeutic intervention.

The tumor suppressor p53 often determines cell fate as a master regulator of stress pathways [10]. In response to free radicals and other DNA-damaging agents, p53 causes a delay in cell cycle progression that allows for DNA repair or induction of cell death via apoptosis. Loss of p53 function can sensitize cells to DNA damage due to impaired DNA repair, but without p53 activity, damaged cells are less likely to undergo apoptosis [11] which may lead to malignant transformation. Many of the downstream effects of p53 are due to transcriptional regulation of targeted genes, and p53 has been shown to regulate expression of miRNAs involved in tumor suppression [12] and cellular senescence [13].

In this study we show that p53 is required for repression of let-7a and let-7b expression in HCT116 colon cancer cells in response to several genotoxic stressors. Furthermore, we show that p53 binds directly to the let-7a3 and let-7b gene enhancer suggesting a mechanism for this repression. In a murine model, we confirm that let-7a and let-7b expression is decreased in radiation sensitive tissues, including bone marrow, lung, and small intestine in wild-type mice, but not in p53 knock-out mice, supporting our hypothesis that p53 is involved in regulation of let-7a and let-7b expression.

## Materials and Methods

### Cell Culture and Treatments

HCT116 p53^+/+^ or p53^−/−^ colon cancer cells (obtained from Dr. B. Vogelstein, Johns Hopkins University in November 2008) were cultured in McCoy's 5A media with 10% FBS, penicillin and streptomycin (100 µg/mL). AG01522 primary fibroblasts (obtained from the Coriell Institute in January 2009) were cultured in F12 media with 20% FBS, penicillin streptomycin. GM5823+hTERT ATM^−/−^ cells (obtained from Dr. T. Pandita, Washington University in March 2009) were cultured in DMEM media with 20% FBS, penicillin streptomycin. ATM null status was confirmed by phosphorylation assays (May 2009). Adhered cells were irradiated using an Eldorado 8 ^60^Co teletherapy unit (Theratronics International Ltd) at dose rates between 150 and 180 cGy/min 1 hour before collection, or with 20 J/m^2^ of UVB light at 254 nm 1 hour before collection [14]. Cells were treated with 100 µM Etoposide (Sigma-Aldrich) or 50 µM H_2_O_2_ (Fisher) 1 hour before collection. Etoposide and H_2_O_2_ treatment levels were determined by previously described methods [3].

### RT-PCR

Cells were lysed using TRIzol reagent (Invitrogen) per manufacturer's instructions., and total RNA was isolated using a standard phenol-chloroform method. RNA concentration was determined in all parts of this study by absorbance at 260 nm using a Nanodrop (NanoDrop Technologies) spectrophotometer. Ten nanograms of total RNA was assayed for specific mature miRNA levels using Taqman miRNA PCR system (ABI) using the standard manufacturer's protocol, and real time-PCR reactions were carried out on an ABI 7500 RT-PCR machine. miRNA levels were normalized to U6 as an internal control.

### Vectors and Transfections

Vectors expressing wild-type p53 (Plasmid 16434), and p53 mutants R273H (Plasmid 16439) and R248W (Plasmid 16437), were obtained from Addgene and contributed by Dr. B. Vogelstein's lab [15]. Vector expressing p53 DD (dominant-negative mutant, Plasmid 9058) was also obtained from Addgene and contributed by Dr. B. Weinburg [16]. HCT116 p53^+/+^ and p53^−/−^ cells were seeded in a 100 mm^2^ plate at 500,000 cells per plate twenty four hours before transfection. Cells were transfected using 10 µL of Lipofectamine 2000 (Invitrogen) and 1 µg of vector mixed with 1 µg of empty vector in 10 mL of Opti-MEM (Invitrogen). Cells were cultured in transfection media for 6 hours then in McCoy's 5A media with 10% FBS. Twenty four hours after the completion of transfection, cells were irradiated as described above.

### Immunoblotting

Following treatment, cells were harvested using RIPA buffer (25 mM Tris•HCl pH 7.6, 150 mM NaCl, 1% NP-40, 1% sodium deoxycholate, 0.1% SDS) supplemented with Complete Protease Inhibitor Cocktail (Roche Molecular Biochemicals) and Halt Phosphatase Inhibitor Cocktail (Pierce). Extract protein concentration was determined using the DC protein assay (Biorad). Thirty micrograms of total protein was subjected to SDS-PAGE on a 4–20% gradient Tris-glycine gel. Protein was transferred to a nitrocellulose membrane using the iBlot transfer system (Invitrogen). Western blots were performed using the following antibodies: anti-p53 (Santa Cruz Biotechnology, antibody #DO-1), anti-P_i_-Ser-15-p53 (Cell Signaling Technology, antibody #9284), and anti-tubulin (Sigma-Aldrich, antibody #T8328).

### Chromatin Immunoprecipitation

ChIP assays were performed as previously described [17]. Immunoprecipitation was performed on irradiated and control samples with anti-p53 antibody (DO-1; Santa Cruz Biotechnology) or mouse IgG2a isotype control (M5409, Sigma-Aldrich). Input and immunoprecipitated DNA samples were purified with a ChIP DNA cleanup kit (Zymo Research). Potential p53 DNA binding sites upstream of let-7a3 and let-7b were identified using Mat Inspector software (Genomatix). Primers were designed using Primerquest software (IDT), primer sequences are listed in Table 1. RT- PCR was performed using SYBR Green PCR Master Mix (ABI) and results were normalized to DNA input controls.

**Table 1:** Primer positions for ChIP.

Primer positions	Primer sequence	p53 sites screened
**Gene OTTHUMG00000150446**		
Forward primer −2034 Reverse primer −1270	5′ TGTAATTCCAGCACATTGGGAGGC 3′ 5′ TATGAATGCCCACTATTCCTGCCC 3′	−1608 and −1913
Forward primer −1098 Reverse primer −393	5′ AGTCTTTCCTGGCACTCACTTAGC 3′ 5′TTTCACACACATCAGAACGGAGGC 3′	−918, −733, −732, and −722
Forward primer −331 Reverse primer +186	5′TTTGTTTGTTTGCCGGCTCC 3′ 5′TTTGTGCCTGCCTCCTCTGC 3′	−83
**Gene OTTHUMG00000030111**		
Forward primer −1645 Reverse primer −1353	5′TCTGAGAGCAAAGACACCTAGAGC 3′ 5′AAAGTGCTGGGATTACAGGCATGAGC 3′	−1529
Forward primer −659 Reverse primer −366	5′TCAAGGAAGGAAAGAACCTTCCCG 3′ 5′AAGGAACTCTGCACTGGAGACC 3′	−452

### Cloning and Luciferase assays

A segment corresponding to bases 46,506,624–46,508,726 on chromosome 22 (Ensembl coordinates) upstream of let-7a3 and let-7b was cloned from human genomic DNA (Promega). The segment was amplified using the Longrange PCR kit (Qiagen), and was ligated into the TopoTA vector (Invitrogen) and subcloned into the expression vectors pGL3 basic, or pGL4.23[*luc2*/minP] vector (Promega). Nucleotides −438–−459, which contain the p53 binding site, were deleted using the QuikChange® II XL Site-Directed Mutagenesis Kit (Stratagene). Cells were transfected with 1 µg of vector mixed with 1 µg of filler DNA (empty pGL3 basic). Cells were treated with IR twelve hours after transfection as described above. Twelve hours after IR cells were collected and luciferase assays were performed on 20 µL of lysate using the luciferase assay system (Promega) as per manufacturer's instructions. Results were normalized to total protein concentration which was determined as described above.

### Irradiation of Mice and Collection and Evaluation of RNA from tissue

Homozygous C57BL/6J or homozygous B6.129-*Trp53^tm1Brd^* N12 (p53 knock-out, Taconic) mice were treated under animal protocols that were reviewed and approved by the National Institute of Health Animal Care and Use Committee IRB in accordance with animal welfare guidelines (approved protocol #H-0083R1) with a total body 2.0 Gy dose using a GammaCell-40® Exactor (Best Theratronics Ltd) 3 hours before sacrifice. Organs were dissected and immediately stabilized by placement in RNAlater (Ambion) per manufacturer's instructions. Approximately 30 mg of tissue was placed in 1 mL of TRIzol reagent (Invitrogen) with lysing matrix D (MP Biomedicals) and homogenized using manufacturer's suggested settings for a FastPrep-24 machine (MP Biomedicals). Following lysis, extraction was performed using a standard phenol-chloroform method. Following extraction, 1 µl of 20 mg/ml glycogen (Fermentas) and 1.5 volumes of 100% ethanol was added to each sample. Total RNA was then purified using RNeasy Mini columns (Qiagen) using standard manufacturer's protocol. RNA concentration was determined and integrity was confirmed by viewing 18S and 28S by denaturing agarose gel electrophoresis. miRNA levels were determined by real time PCR and normalized using the endogenous small RNA control snoRNA 202 (ABI). mRNA expression for BAX and PUMA were assessed using the High Capacity cDNA Reverse Transcription kit (ABI) as per manufactures instructions. RT-PCR reactions were performed using 20 ng of cDNA, 125 nM primers for Bax and PUMA were used (listed below) and were normalized to 18S.

### Bax

Forward 5′–ATGCGTCCACCAAGAAGCTGAG-3′

Reverse 5′-CCCCAGTTGAAGTTGCCATCA-3′

### PUMA

Forward 5′-TCCTCAGCCCTCCCTGTCAC-3′

Reverse 5′-CCATTTCTGGGGCTCCAGGA-3′

### Statistics

All values represent mean of three experiments unless stated otherwise. Error bars represent standard deviation. p-value determined by Student's *t*.

## Results

### Exposure to radiation and oxidative stress decreases let-7a and let-7b in a p53 dependent mechanism

**Figure 1:**
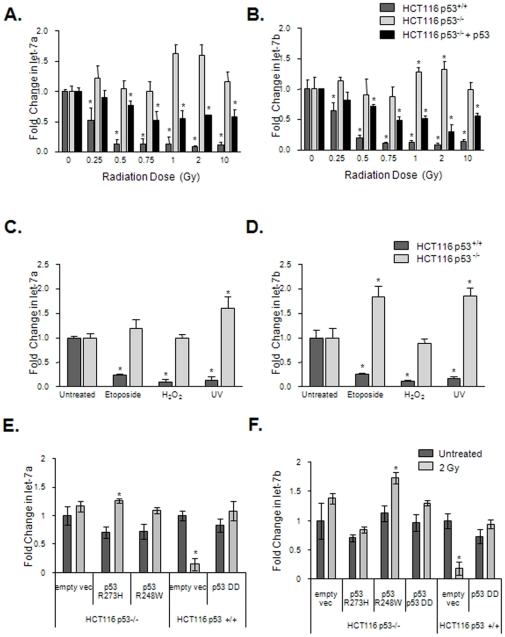
let-7a and let-7b expression are altered *in vitro* in response to genotoxic stress in a p53 dependent manner. HCT 116 p53^+/+^ cells, HCT116 p53^−/−^ cells, and HCT116 p53^−/−^ cells transfected with vector expressing exogenous p53 were collected 1 hr after irradiation and evaluated by RT-PCR for expression of let-7a (A) and let-7b (B). Expression of both species decreased with radiation in the p53^+/+^ cells and p53^−/−^ cells transfected with exogenous p53, but not in the p53^−/−^ cells. Cells were then treated with etoposide, H_2_O_2_, or UV radiation and assessed for let-7a (C) and let-7b (D). These genotoxic stressors similarly resulted in a decrease of both let-7 species that was observed only in the p53^+/+^ cells. Vectors expressing p53 R273H, p53 R248W, or control empty vector were transfected into HCT116 p53^−/−^ or p53^+/+^ cells 24 hours prior to irradiation to 2 Gy. One hour after irradiation, cells were collected. Real-Time PCR for let-7a (E) or let-7b (F) was performed. Transfection of mutant p53 into HCT116 p53^−/−^ cells did not rescue repression of let-7a and let-7b after radiation. * denotes p<0.05.

let-7a and let-7b account for about 60% of overall let-7 expression in HCT116 cells [18] and about 70% in AG01522 cells [3]. Furthermore only let-7a and let-7b have been reported to decrease at both high and low doses of radiation, thus we chose to focus on those two members of the let-7 family. To determine if this decrease is dependent on p53, HCT116 p53^+/+^ and p53^−/−^ cells were exposed to radiation doses ranging from 0.25 to 10 Gy, and total RNA was collected after 1 hour and analyzed by real-time PCR for mature miRNA (Fig. 1A and B). In the p53^+/+^ cells, both let-7a and let-7b expression decreased significantly even at the lowest radiation dose administered. In contrast, let-7a and let-7b expression did not decrease in the HCT116 p53^−/−^ cells. A similar decrease in let-7a and let-7b expression was also observed following exposure to H_2_O_2_, etoposide, and UV radiation and this decrease required p53 (Fig. 1C and D). These results suggest that p53 plays an important role in stress-induced miRNA expression changes and is required for the observed decrease in let-7 expression signaled by DNA damage.

To confirm these results the HCT116 p53^−/−^ cells were transfected with either exogenous wild-type or mutant p53 genes. Transient expression of wild-type p53 restored the decrease in let-7a and let-7b expression following exposure to radiation (Fig. 1A and B). However, expression of the p53 DNA-binding mutants R273H and R248W or a dominant-negative p53 in HCT116 p53^−/−^ cells (Fig. 1E and F) did not rescue this repression. Dominant-negative p53 is known to prevent tetramerization of wild-type p53 thereby preventing its activation [16], and transfection of dominant-negative p53 into HCT116 p53^+/+^ cells prevented repression of let-7a and let-7b (Fig. 1E and F). The results of these experiments further demonstrate that functional p53 is required for the generation of radiation-induced alterations in let-7a and let-7b.

**Figure 2:**
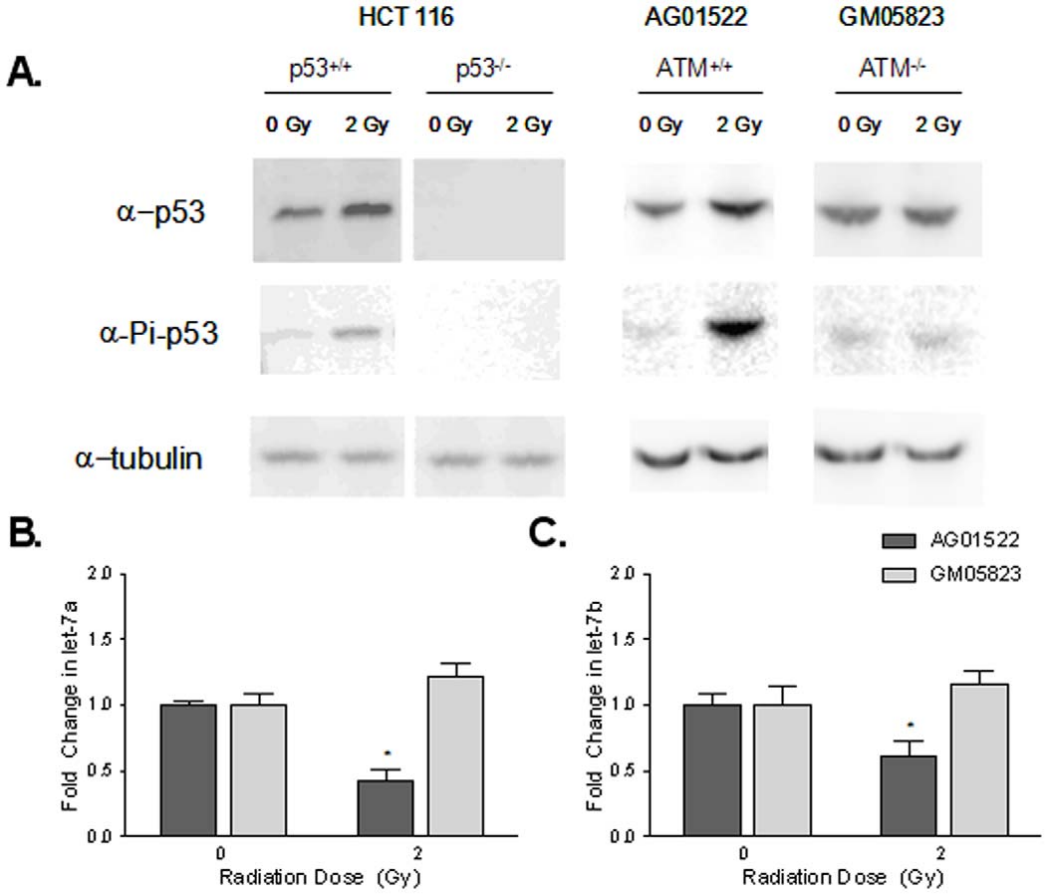
Repression of let-7a and let-7b is dependent on ATM phosphorylation of p53. HCT116 p53^+/+^, p53^−/−^ cells, ATM^+/+^ (AGO1522) and ATM^−/−^ (GM05823) fibroblasts were collected 1 hour after 2 Gy irradiation. Expression of p53, phosphorylated (ser-15) p53, or tubulin was assessed by western blot (A). Total and phosphorylated p53 were increased after radiation in the p53^+/+^ and ATM^+/+^ cells, but not the p53^−/−^ or ATM^−/−^ cells. ATM^+/+^ and ATM^−/−^ fibroblasts were also collected after irradiation and expression of let-7a (B) and let-7b (C) decreased in the ATM^+/+^cells but not in ATM^−/−^ cells suggesting that ATM-dependent p53 activation is necessary for radiation-induced changes in let-7 expression.* denotes p<0.05.

Cellular exposure to radiation induces the activation of the ATM kinase, which directly phosphorylates p53 at serine-15 and promotes phosphorylation at other sites, leading to p53 stabilization and transcriptional regulation. Western blots confirmed an increase in expression of both total p53 and serine-15 phosphorylated p53 following a 2 Gy dose of radiation in both HCT116 p53^+/+^ cells and AG01522 ATM^+/+^ primary human fibroblasts (Fig. 2A). However, no increase in total p53 or phosphorylated p53 was detected in HCT116 p53^−/−^ cells or in an ATM^−/−^ human fibroblast cell line (GM05823) (Fig. 2A). Furthermore, after irradiation let-7a and let-7b expression decreased in ATM^+/+^ fibroblasts but not ATM-deficient fibroblasts (Fig. 2B and C) suggesting that ATM-dependent p53 activation is necessary for radiation-induced changes in let-7 expression.

### p53 interacts directly with the let-7a3 and let-7b enhancer

**Figure 3:**
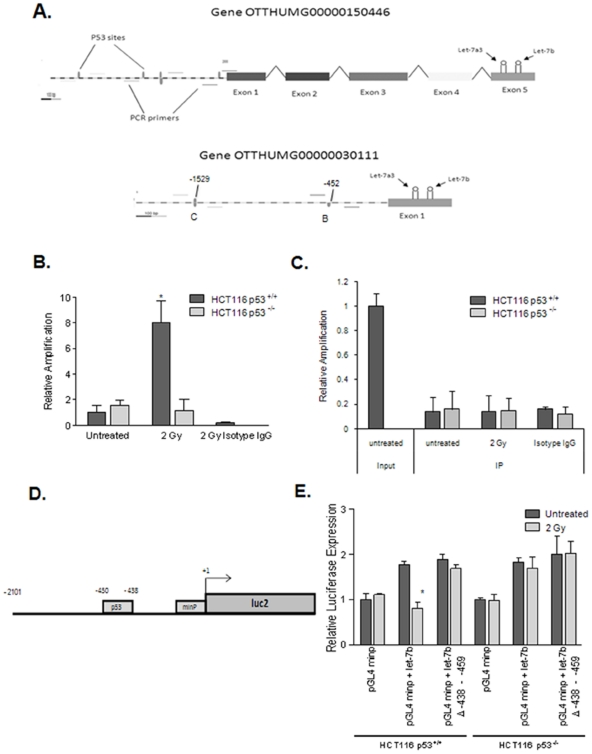
p53 interacts with the let-7a3 and let-7b gene enhancer. (A) PCR primers were designed to potential p53 binding sites upstream of let-7a3 and let-7b gene overlapping transcripts OTTHUMG00000030111 and OTTHUMG00000150446. HCT 116 cells were irradiated, fixed and p53 was immunoprecipitated using anti-p53 antibody. Real-time RT-PCR was then performed which revealed binding in the p53^+/+^ cells after radiation but not in the p53^−/−^ cells (B). All results were normalized to input DNA. (C) Human genomic DNA upstream of let-7a3 and let-7b containing the p53 binding site was cloned upstream of luciferase in the vectors pGL3 basic or pGL4.23[*luc2*/minP]. Each of these constructs was transfected into HCT116 p53^+/+^ cells. Twenty-four hours after transfection, lysates were collected and assayed for luciferase activity (D). The active pGL4.23[luc2/minP] clone was transfected into HCT116 p53^+/+^ and p53^−/−^ cells that were irradiated to 2 Gy and assayed for luciferase expression. Although no change in luciferase expression was noted with transfection of the minimal promoter alone, the addition of the enhancer element resulted in suppression of luciferase expression in the HCT116 p53^+/+^ cells but not the p53^−/−^ cells (E). * denotes p<0.05.

Like many miRNA, let-7a is expressed from multiple locations in the genome. However, let7-a3 and let-7b are clustered within approximately 900 bps on chromosome 22 . Therefore this locus was selected to probe for a p53 interaction. Both miRNA precursors overlap with novel Vertebrate Genome Annotation (VEGA) genes OTTHUMG00000030111 (HGNC symbol MIRLET7BHG) and OTTHUMG00000150446. Scanning of the DNA sequence upstream of these genes with Genomatix MatInspector software revealed several potential p53 binding sites (Fig. 3A). ChIP was performed with p53 antibody and PCR primers bracketing these sites to probe for an interaction with p53. One p53 binding site at location −450 to −438 upstream of the transcription start site OTTHUMG00000030111 demonstrated an interaction with p53 (Fig. 3A). RT-PCR of immunoprecipitated samples indicated an interaction of this locus with p53 that occurred after exposure to 2 Gy (Fig. 3B). These data show that following irradiation, p53 interacts with DNA upstream of the let7-a3 and let-7b genes suggesting a mechanism for p53-dependent radiation-induced repression of let-7a and let-7b expression.

The region of DNA upstream of let-7a3 and let-7b that contains the p53 binding site was cloned upstream of luciferase in the vectors pGL3 basic, and pGL4.23[*luc2*/minP] (Fig. 3C). Only the pGL4 construct showed positive luciferase expression over controls when transfected into HCT116 cells (Fig. 3D), suggesting that this region is not a functional promoter on its own, but can act as an enhancer of transcription. Following transfection with this enhancer element, cells were treated with a 2 Gy dose of ionizing radiation. At 12 hours post-radiation, luciferase expression luciferase activity was no longer enhanced over empty vector control in HCT116 p53^+/+^ cells but remained elevated in p53^−/−^ cells (Fig. 3E), or following deletion of the predicted p53 binding site. This result further supports our assertion that the interaction of p53 with the DNA element is important for repression of let-7a and let-7b following irradiation.

### Exposure to radiation and oxidative stress decreases let-7a and let-7b in a p53 dependent mechanism

**Figure 4:**
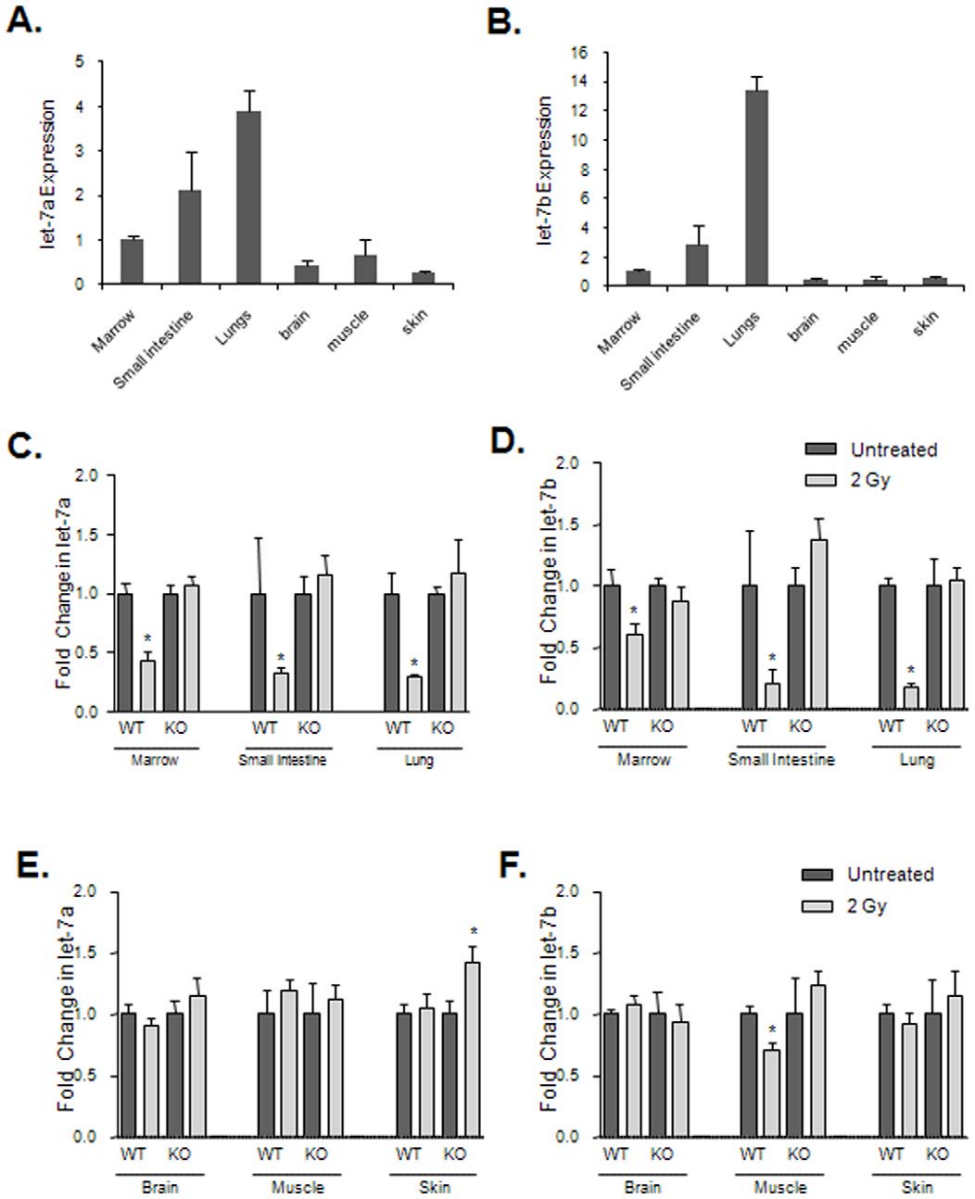
Altered let-7a and let-7b expression *in vivo* in response to genotoxic stress is p53 dependent. Total RNA from was collected from tissues of C57BL/6J mice and assayed for let-7a (A) or let-7b (B) by real-time PCR. Homozygous C57BL/6J or homozygous B6.129-Trp53tm1Brd N12 (p53^−/−^, Taconic) mice were exposed to 2 Gy total body radiation. Tissues were collected 3 hours after irradiation and radiosensitive tissues including bone marrow, small intestine, and lung were assessed for let-7a (C) and let-7b (D) which demonstrated decreased expression in the p53^+/+^ but not the p53^−/−^ tissues. This p53-dependant reduction was not seen in more radiation insensitive tissues such as brain, muscle, and skin for either let-7a (E) or let-7b (F). Results are the average of values from 2 mice. * denotes p<0.05.

let-7a and let-7b expression levels were assayed in several well established radiation sensitive tissues (small intestine, lung, and bone marrow), and radiation resistant tissues (brain, muscle, and skin) in C57BL/6J mice (Fig. 4A and B). Basal let-7 expression is greater in radiation sensitive tissues, especially lung. The *in vivo* response of let-7 to DNA damage was determined by treatment of C57BL/6J wild-type and p53 knock-out mice with 2.0 Gy total body irradiation (TBI). let-7a and let-7b expression was significantly reduced in radiation sensitive tissues in the wild-type mice (Fig. 4C and D). In contrast, radiation resistant tissues from the wild-type mice did not exhibit a decrease in let-7 expression (Fig. 4E and F). Expression of let-7 remained either unchanged or increased in all tissue types collected from p53 knock-out mice. These *in vivo* findings confirm our previous *in vitro* results, and support the hypothesis that p53 plays an important role in radiation-induced changes in let-7a and let-7b expression.

**Figure 5:**
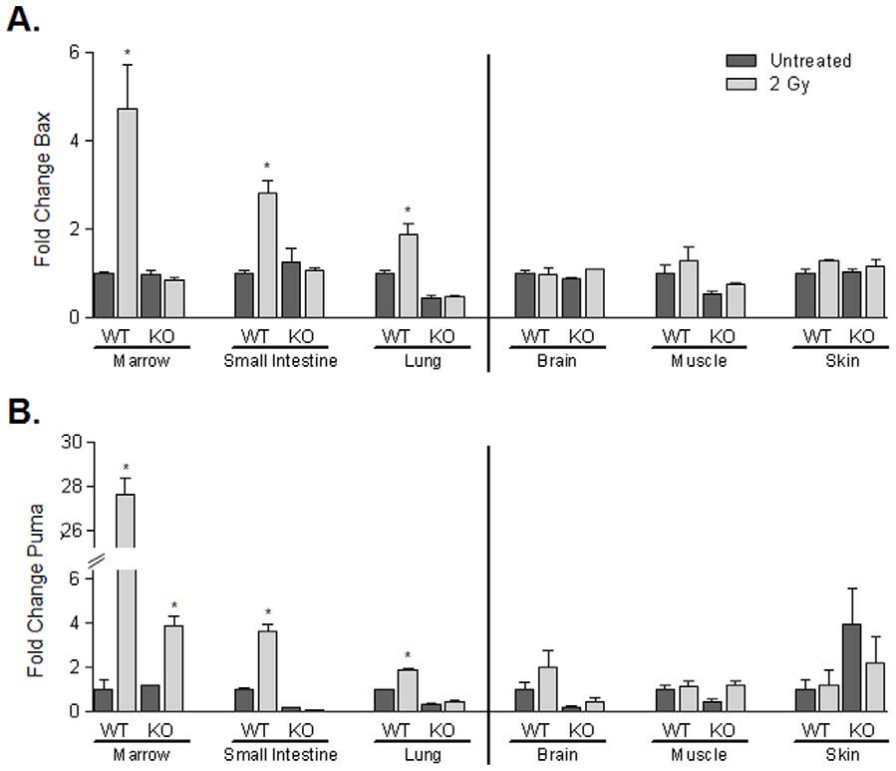
Transcription of other p53 regulated genes such as Bax, PUMA correlates with let-7a and let-7b expression after irradiation. Homozygous C57BL/6J (p53 wild-type) or homozygous B6.129-Trp53tm1Brd N12 (p53 knock-out, Taconic) mice were exposed to 2 Gy total body irradiation and were assessed for expression of Bax (A) and PUMA (B). Bax and PUMA exhibit increased expression in p53 wild-type radiation sensitive tissues which is similar to the let-7 expression pattern, but do not exhibit increased expression in radiation insensitive tissues, or tissues from p53 knock-out mice (other transcription factors mediate minimal expression of Puma in the absence of p53). All results represent the average of values from two mice. * denotes p<0.05.

The radiation responsiveness of let-7a and let-7b expression inversely correlates with the transcription of other p53-regulated target genes across mouse tissues. The mRNA levels of the p53 regulated genes Bax and PUMA were analyzed by RT-PCR in both radiation-sensitive and radiation-resistant tissues. Similar to the response of let-7 expression, both Bax and PUMA showed a much greater change in expression in radiation-sensitive tissues compared to radiation- resistant tissues (Fig. 5A and B, respectively) which may suggest that let-7a and let-7b regulation by p53 may be associated with the p53 mechanisms that induce apoptosis.

## Discussion

Several previous studies have shown that the let-7 family of miRNA is repressed following exposure to radiation in multiple cell lines [2], [3], [19], [20], [21]. These radiation-induced reductions in let-7a and let-7b expression could be a part of the cellular response to oxidative stress or may be due to DNA damage-associated signaling pathways. We hypothesized that, since p53 is activated by both oxidative stress and DNA damage, p53 could be involved in the mechanism underlying the observed let-7 expression changes.

Our results confirm a radiation-induced decrease in let-7 expression in both HCT116 p53^+/+^ colon cancer cells and ATM^+/+^ fibroblasts. However, we show that let-7a and let-7b expression does not decrease, and may in fact be enhanced, in both HCT116 p53^−/−^ cells and ATM^−/−^ fibroblasts suggesting that ATM-mediated stabilization of p53 plays an important role in repressing let-7a and let-7b expression in response to radiation exposure. Finally, let-7a and let-7b repression is rescued through expression of exogenous wild-type p53 in HCT116 p53^−/−^ cells.

To further study this interaction between p53 and let-7 expression, an *in silico* analysis was performed which identified a possible p53 binding site approximately 450 bp upstream of the let-7a3/let-7b gene. ChIP experiments confirmed that an interaction between p53 and the proposed binding site occurs in response to radiation-induced cell stress. Also, the p53 DNA binding site was able to repress luciferase expression in reporter-based assays in HCT116 p53^+/+^ cells but not p53^−/−^ cells. Although p53 utilizes multiple mechanisms to promote repression, this binding suggests that p53 may directly mediate expression of the let-7a and let-7b genes [22]. A recent study has shown that p53 can interact directly with the miRNA processing enzyme Drosha [23], and it is therefore possible that this may also play a role in the p53-mediated repression of the let-7 family, in addition to transcriptional regulation.

Radiation-induced repression of let-7a and let-7b expression is also observed in mice that have undergone total body irradiation (TBI) to 2 Gy. This repression appears to be tissue specific since a decrease in let-7a and let-7b expression was observed in radiation sensitive tissues such as lung, bone marrow, and small intestine, but either no change or an enhancement of expression was observed in radiation resistant tissues such as brain, skin, or muscle. This differential response in let-7 expression closely mimics the difference we observed between HCT116 p53^+/+^ and p53^−/−^ cells suggesting a similar mechanism might be responsible. In support of this hypothesis is the fact that radiation sensitive tissues typically undergo an apoptotic cell death, which involves p53, while radiation resistant tissues typically undergo a p53-independent mitotic cell death. Furthermore, we show that this response also correlates with p53-regulated expression of the pro-apoptotic genes Bax and PUMA. Taken together, these data suggest that a tissue-specific p53 response underlies the changes in let-7a and let-7b expression we have observed.

let-7a and let-7b expression is higher in radiation sensitive tissues. Studies have shown that over-expression of let-7 increases sensitivity of cells to radiation [2] and cisplatin [24]. Therefore it is logical to suggest that higher expression of let-7 may contribute to radiation sensitivity. The mechanism by which this action occurs should be further studied. In addition to sensitizing to cytotoxic agents, it has been previously observed that let-7 can significantly slow tumor growth *in vivo* [6] and suppress stem cell characteristics in tumors [25]. These observations taken together display significant potential of let-7 mimics as adjuvant cancer therapeutics.
