# Correction: Cellular Stress Induced Alterations in MicroRNA let-7a and let-7b Expression Are Dependent on p53

**DOI:** 10.1371/annotation/5c1dd48c-38e4-41ed-b827-748f62e28365

**Published:** 2012-04-23

**Authors:** Anthony D. Saleh, Jason E. Savage, Liu Cao, Benjamin P. Soule, David Ly, William DeGraff, Curtis C. Harris, James B. Mitchell, Nicole L. Simone

A previous version of the manuscript was mistakenly published. The correct text can be viewed here: http://www.plosone.org/corrections/pone.0024429.text.cn.doc

