# Correction: Cellular Stress Induced Alterations in MicroRNA let-7a and let-7b Expression Are Dependent on p53

**DOI:** 10.1371/annotation/9b0f3eff-0376-43ca-b357-adfd8fc9ac7a

**Published:** 2012-04-23

**Authors:** Anthony D. Saleh, Jason E. Savage, Liu Cao, Benjamin P. Soule, David Ly, William DeGraff, Curtis C. Harris, James B. Mitchell, Nicole L. Simone

A previous version of the manuscript was mistakenly published. A note linking to a correct version of the article can be found on the article page. Text was removed from the Abstract. A sentence was removed from the first paragraph in the Results and three sentences were added to the fourth paragraph in the Results. Text was added to the second and fourth paragraphs in the Discussion and text was modified in the third paragraph in the Discussion. The text for the legend of Figure 3 was also modified. The figures themselves were published correctly. 

